# Supplementary figures and images for: Remarkable genomic diversity among Escherichia isolates recovered from healthy chickens
Source: PeerJ. 2022 Mar 1;10:e12935. doi: 10.7717/peerj.12935 (PMC8896058; doi:10.7717/peerj.12935)

**(A) ClermonTyping Phylogroups**

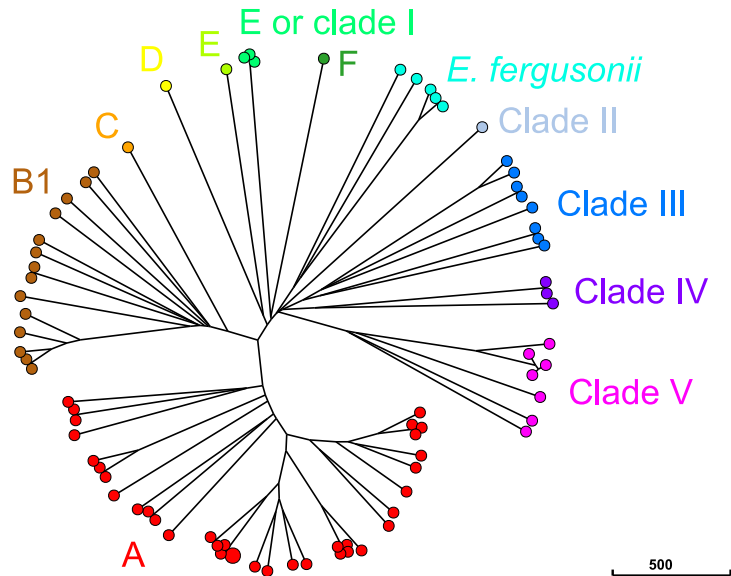

**(B) Collection Month**

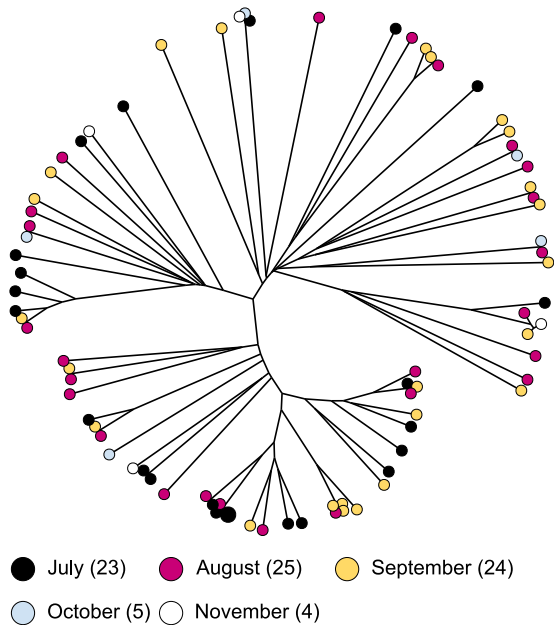

Supplement: Supplemental Information 1 — The same tree is shown with nodes colored by (A) predicted phylogroup according to the program ClermonTyping, and (B) month in which the fecal sample was collected. Isolates cluster strongly with their predicted phylogroups, and isolates from all groups were recovered throughout the experiment. [file peerj-10-12935-s001.pdf]
